# Harmonized and high-quality datasets of aerosol optical depth at a US continental site, 1997–2018

**DOI:** 10.1038/s41597-021-00866-2

**Published:** 2021-03-11

**Authors:** Evgueni Kassianov, Erol Cromwell, Justin Monroe, Laura D. Riihimaki, Connor Flynn, Jaime Barnard, Joseph J. Michalsky, Gary Hodges, Yan Shi, Jennifer M. Comstock

**Affiliations:** 1 Pacific Northwest National Laboratory, Richland, WA USA; 2 Cooperative Institute for Mesoscale Meteorological Studies, University of Oklahoma, Norman, OK USA; 3 National Oceanic and Atmospheric Administration, National Severe Storms Laboratory, Norman, OK USA; 4 Cooperative Institute for Research in the Environmental Sciences, Boulder, CO USA; 5 National Oceanic and Atmospheric Administration, Global Monitoring Laboratory, Boulder, CO USA; 6 School of Meteorology, University of Oklahoma, Norman, OK USA; 7 University of Nevada, Reno, NV USA

**Keywords:** Atmospheric dynamics, Design, synthesis and processing

## Abstract

Aerosol optical depth (AOD) characterizes the aerosol burden in the atmosphere, while its wavelength dependence is a sign of particle size. Long-term records of wavelength-resolved AOD with high quality and suitable continuity are required for climate change assessment. Typically, climate-related studies use AOD products provided by several, and perhaps different, ground-based instruments. The measurements from these instruments often have different accuracy and temporal resolution. To preserve the advantages of these products (high quality) and to reduce their disadvantages (patchy records), we generate a merged dataset obtained from four instruments deployed at a US continental site in which a nearly-continuous AOD record is found at two wavelengths (500 and 870 nm) with high quality and high temporal resolution (1-min) for a 21-yr period (1997–2018). The combined dataset addresses: (1) varying data quality and resolution mismatch of the individual AOD records, and (2) the uncertainty of the merged AOD and its relevance for user-specified needs. The generated dataset will be beneficial for a wide range of applications including aerosol-radiation interactions.

## Abstract (summary)

**Table:** 

Measurement(s)	aerosol optical depth
Technology Type(s)	atmospheric observatory system
Factor Type(s)	year of data collection
Sample Characteristic - Environment	atmosphere
Sample Characteristic - Location	State of Oklahoma

Machine-accessible metadata file describing the reported data: 10.6084/m9.figshare.13256699

## Background & Summary

The aerosol optical depth (AOD) is the key parameter characterizing atmospheric aerosol and has several important climate-related influences due to the complex interplay of aerosol particles with radiation and clouds^1–3^. There is a growing awareness that both the temporal and spatial variability of AOD^4,5^ may have a significant effect on the aerosol radiative forcing^6,7^ and thus on the direction and magnitude of climate change^8,9^. Therefore, long-term records of AOD with high quality and temporal continuity at climate-important locations are required to draw firm conclusions about the importance of this variability in forcing the climate change. These records are provided by several established operational AOD networks, including the National Oceanic and Atmospheric Administration surface radiation budget (SURFRAD) network^10^, the Skyradiometer Network (SKYNET)^11^ and the National Aeronautics and Space Administration (NASA) Aerosol Robotic Network (AERONET)^12^ with two associated sub-networks^13,14^. Observational studies typically involve several instruments with different designs and operational techniques to measure AOD^15–17^, and thus individual AODs from different instruments will likely have different continuity and accuracy. In view of increasing demands for continuous long-term records of AOD with high quality, the development of integrated datasets of AOD is now crucial^18,19^. Henceforth, the term “individual” will denote an AOD record obtained from a particular instrument. Since individual AODs have different advantages and shortcomings, preserving the former and reducing the latter is a well-known challenge of such measurement integration.

There are several sites with collocated ground-based instruments for AOD measurements^15,16^. Here we focus on multi-year time series of AOD available at the mid-continental Southern Great Plains (SGP) Central Facility (CF; 36.605°N, 97.485°W) supported by the Office of Science of the U.S. Department of Energy (DOE) as part of the Atmospheric Radiation Measurement (ARM) user facility^20^. These time series are provided for more than two decades by four collocated ground-based instruments^21,22^. Two of them, the so-called Multifilter Rotating Shadowband Radiometers (MFRSRs), are sensors with hemispherical receptors that are periodically shaded by rotating bands^23^. Two other instruments, the so-called Normal Incidence Multifilter Radiometer (NIMFR)^23^ and Cimel Sunphotometers (CSPHOT), are sensors with a sun-pointing design. The CSPHOT is part of the AERONET and the CSPHOT operation differs significantly from the operation of the MFRSR and NIMFR. The different design of these instruments and concomitant data processing schemes define the instrument-dependent continuity, quality and resolution of the corresponding AOD products.

The four individual AOD records outlined above create a unique opportunity for generating a multi-year dataset of a single “combined” AOD record with high quality, enhanced continuity and coverage. Here we explore this opportunity by addressing two major challenges associated with: (1) varying data quality and the time resolution mismatch of the individual records, and (2) the uncertainty of the combined AOD and its relevance for user-specified needs. Our initial efforts are focused on generating a nearly-continuous combined AOD at two wavelengths (500 and 870 nm) with high quality and fine temporal resolution (1-min) for a 21-yr period (1997–2018). We demonstrate that the combined AOD as compared to its individual components has an extended temporal coverage (e.g., up to 40% relative to the CSPHOT AOD), while uncertainty of the combined AOD does not exceed the prescribed uncertainties (0.01–0.02) of the individual AODs for the majority of cases (about 90% of time). Thus, the generated dataset with combined AOD preserves the advantages of the individual AODs (high data quality) and reduces their disadvantages (gaps in AOD time series).

We expect that the generated dataset will receive considerable attention from researchers working on a wide range of climate-related multi-disciplinary projects involving both model simulations and data analysis. For example, long-term AOD records with seasonal and interannual variability are required for improved understanding of trends in aerosol loading driven by natural and anthropogenic emissions^4,24^ and the corresponding aerosol-induced radiation trends^25,26^. Alternatively, high-quality AOD obtained at high temporal scales are needed for process-oriented studies^27,28^ and radiative closure experiments with the main goal to determine the precision with which a radiative transfer model can predict both direct and diffuse irradiances^29,30^. Finally, information on the uncertainty of the measured AOD is critical for the aerosol radiative forcing and its error estimation^31–33^.

## Methods

### General approach

The AOD measurements from the four instruments outlined in the previous section can be considered as a group of records with overlapping intervals. These records collectively provide an opportunity to obtain a combined AOD with an extended continuity in comparison with those offered by measurements from a single instrument. Here we consider this opportunity and demonstrate how the combined AOD and its uncertainty can be obtained using a straightforward and intuitively understandable approach. We demonstrate, for example, that a single parameter, which describes spread between individual AODs, facilitates assessment of this uncertainty and the level of agreement between individual records for the overlapping periods. Such assessment is essential for generation of long-term AOD records with user-specified data quality.

**Fig. 1:**
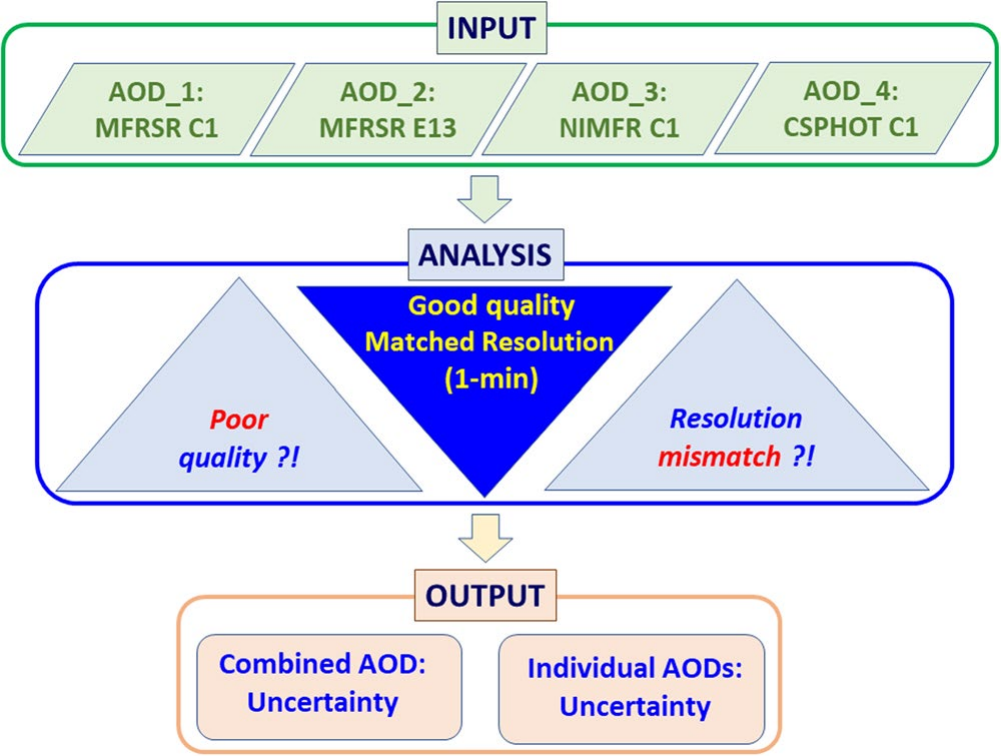
Schematic diagram summarizing the framework for generating a combined AOD with extended continuity and user-specified data quality. The diagram illustrates a connection between three main sections: (1) involvement of individual AODs measured by four collocated instruments (top part of diagram), (2) examination of data quality and resolution consistency (central part of diagram), and (3) determination of the combined AOD and its four individual components with good quality and matched resolution (bottom part of diagram). Uncertainty of the combined AOD and the main statistics describing the level of agreement between these components are also established. See indicated text sections for details of each component (Input, Analysis, Output). Note that MFRSR C1 and MFRSR E13 are identical instruments sitting next to each other and labels C1 and E13 denote specific instruments (Table 1).

We start with a broad description of our approach with three main components (Fig. 1) and then provide the corresponding details in subsequent sections. The first main component deals with acquiring AOD records from the four ground-based collocated instruments with different design, operation and temporal resolution (“Instrument-dependent AODs” section). The second component defines AODs with good quality (“Data Quality Assessment” section) and harmonized 1-min temporal resolution (“Resolution Matching” section). The third component utilizes the defined AODs and calculates the combined AOD and its variability (“Combined and Individual AODs and Their Uncertainties” section). The variability assessment is complemented by traditional pairwise comparisons of the individual AODs.

### Input: Instrument-dependent AODs

The MFRSR measures total and diffuse solar irradiances at five nominal wavelengths (0.415, 0.5, 0.615, 0.673 and 0.87 μm) from which AODs may be derived. These quantities are acquired from both the shaded and unshaded hemispheric measurements and are used to calculate the direct sun irradiance. Since the MFRSR has a quasi-Lambertian receiver, corrections for the imperfect cosine response are required. These corrections to the cosine response are provided by the SGP calibration facility independently for the two MFRSRs considered here. Also, independent in-field calibrations based on Langley regression for extraterrestrial response are performed for these instruments. The direct sun irradiance, in turn, is used to derive the corresponding wavelength-dependent AOD. The calibration uncertainty of the MFRSR-derived AOD is estimated as ± 0.005 + 0.01/*m*, where *m* is the optical air mass relative to the path in the zenith direction^34^.

The NIMFR has the same electronics and receiver as the MFRSR. However, in contrast to the MFRSR, the NIMFR has a sun-pointing design that directly measures the direct normal irradiance. Therefore, corrections for cosine response are not required. The NIMFR has a field-of-view (FOV) with a full angular width of 5.7° and measures direct sun irradiance at five nominal wavelengths (0.415, 0.5, 0.615, 0.673 and 0.87 μm) simultaneously to calculate AOD. The wavelength-dependent AOD is derived from the NIMFR-measured direct sun irradiance. In-field calibrations based on Langley regression are applied to the NIMFR data in a fashion similar to the MFRSR measurements. The calibration uncertainty of the NIMFR-derived AOD is the same as the calibration uncertainty of the MFRSR-derived AOD^34^.

The CSPHOT with a sun- and sky-tracking design and 1.2° FOV measures both direct sun irradiance and sky radiance. The AOD is derived at eight wavelengths (0.34, 0.38, 0.44, 0.5, 0.675, 0.87, 1.02, and 1.64 μm) from the measured direct sun irradiance. The CSPHOT calibration involves intercomparison with reference instruments located at the Goddard Space Flight Center (GSFC). The reference instruments are Langley calibrated at the Mauna Loa Observatory on the island of Hawaii. The CSPHOT GSFC calibration is performed approximately every 6 to 12 months. The calibration uncertainty of AOD obtained from this field instrument is approximately 0.01–0.02^35^. It should be mentioned that the CSPHOT deployed at the ARM SGP CF is part of the AERONET network^12^, and thus AERONET processes the CSPHOT data for ARM. ARM takes the processed AERONET data and ingests them into the ARM standard format ‘and archived for potential users. The sun- and sky-tracking measurement design of the CSPHOT is responsible for the relatively coarse temporal (about 10–15 min) resolution of the CSPHOT AOD in comparison with the higher temporal (20 sec) resolution of the MFRSR and NIMFR AODs.

### Analysis: Data quality assessment

AODs generated from both MFRSRs and NIMFR measurements include Quality Control (QC) variables. These variables characterize the data as “good”, “suspect” or “incorrect”. The term “incorrect” indicates that the AOD values are inaccurate and should not be used, while the term “suspect” specifies that the AOD values are exhibiting some indication of an underlying issue and additional screening is required. For this study, we use only the “good” data as indicated by the automated QC tests.

AODs generated from the CSPHOT measurements are controlled by the AERONET-established data quality and analysis. There are three data quality levels, which characterize “unscreened” (Level 1.0), “cloud-screened and quality controlled” (Level 1.5) and “quality-assured” (Level 2.0) data. In addition to the automated near-real-time quality control analysis, the data quality is manually inspected by the ARM CSPHOT instrument mentors both daily and weekly^35^. It should be mentioned that the “quality-assured” (Level 2.0) data are generated after the post field calibration. The most recent AERONET Version 3 (V3) quality control procedure^36^ has improved cloud screening in comparison with its previous counterpart^37^, thus we use this version (V3 Level 2.0 AOD) in our subsequent analysis.

### Analysis: Resolution matching

After passing the automated QC tests outlined in the previous section, the “good” AODs generated with 20-sec temporal resolution from both MFRSRs and NIMFR measurements are resampled to produce the corresponding 1-min counterparts. The 20-sec datapoints are used to calculate the 1-min average if their AOD values are within the specified spectrally-independent range (from 0 to 1). In other words, these datapoints should be positive and not exceed the maximum permissible AOD of 1.0. Note that this value is a reasonable limit to indicate cloud-contaminated data as the largest value of the daily-averaged AOD at 0.5 μm wavelength does not surpass 0.6 during multi-year ground-based observations performed previously at the ARM SGP CF^21,22^. Moreover, these observations have not documented any statistically significant AOD trends. A datapoint is likely cloud-contaminated if its value is in excess of the maximum permissible AOD.

The “quality-assured” (Level 2.0) instantaneous AERONET AODs are generated with lower (about 10–15 min) temporal resolution from the CSPHOT measurements. Thus, the 1-min averaging is not performed for the CSPHOT AODs. Instead, 1-min intervals are found where the instantaneous CSPHOT AODs are available. They are considered as 1-min averages for these intervals. Clearly, the number of these 1-min intervals with instantaneous CSPHOT AODs is substantially smaller than those where the 1-min averages of AOD are calculated from the corresponding 20-sec MFRSR/NIMFR datapoints. Overall, the resolution matching considered here provides four individual AOD records (MFRSR C1, MFRSR E13, NIMFR and CSPHOT) with the same 1-min resolution.

### Output: Combined and individual AODs and their uncertainties

The combined AOD (also referred to as the best estimate AOD) at a given time is calculated as the average of overlapped individual AODs. In other words, the combined AOD is the sum of overlapped individual AODs divided by their number. These AODs represent the same 1-min interval. For the ground-based instruments considered here, this number can change from 2 (AODs are available from two instruments only) up to 4 (AODs are available from all instruments). If only one individual AOD is available for a given time (i.e., AODs from other instruments are unavailable), then the combined AOD is the individual AOD. Records of individual AODs have distinct continuities for the site considered here, thus the calculation of the combined AOD involves a time-dependent number of these AODs. The outlined temporal dependence should be considered properly during potential calculations of the combined AOD for other sites with different sets of accessible ground-based instruments.

**Fig. 2:**
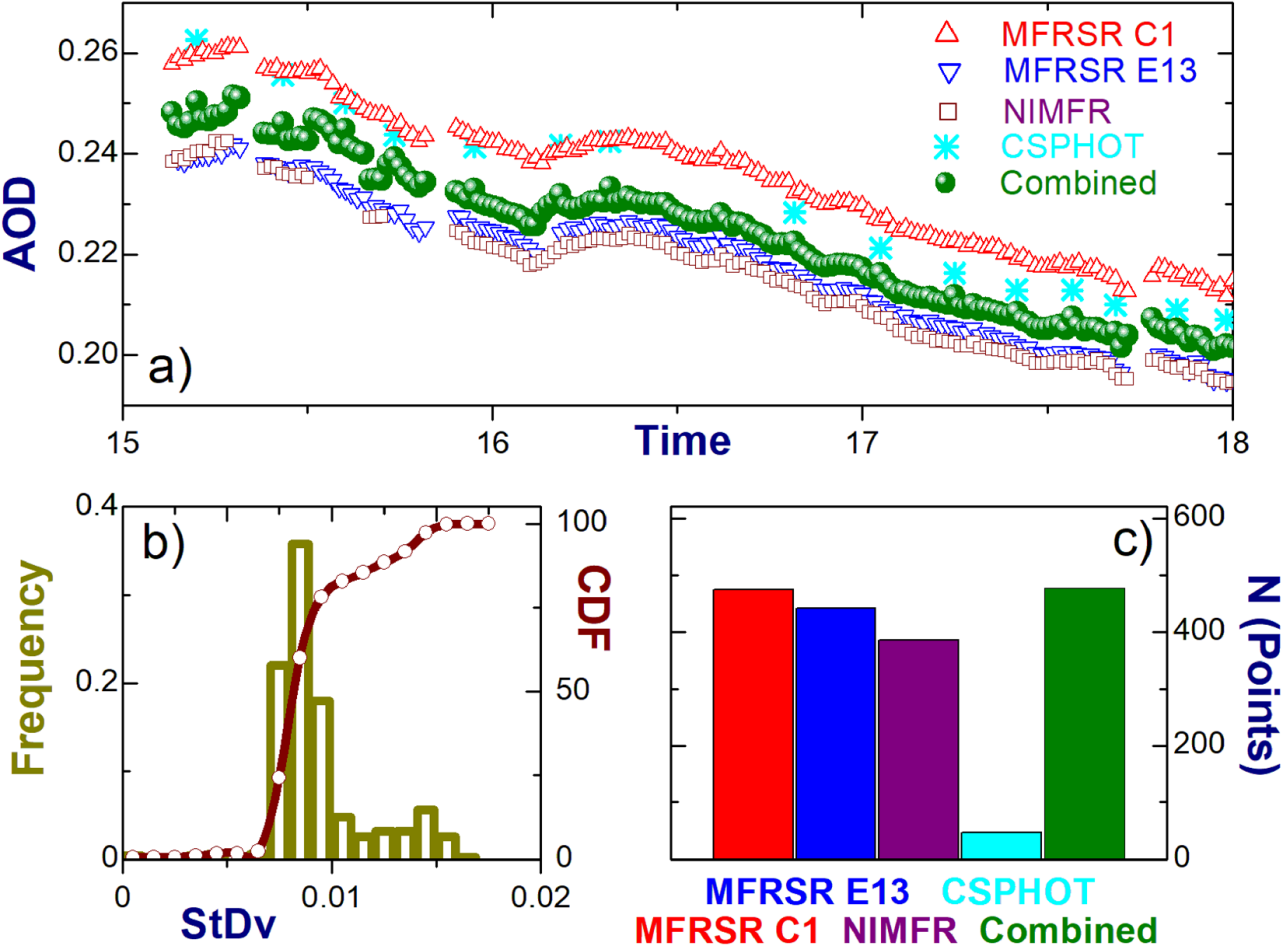
Demonstration of how to combine the individual AODs in a complimentary way for a given day (May 11, 2008). (**a**) diurnal changes of the individual and combined AODs. To ease visual comparison of these AODs, we “zoom-in” on their diurnal changes by selecting a narrow (3-h) temporal window. (**b**) normalized frequency and cumulative distribution function (CDF) of standard deviation (StDv) of the combined AOD calculated for the given day, (**c**) the number of points (time instances) of the individual and combined AODs calculated for overlapped periods.

Figure 2 shows an example of the combined AOD at 0.5 μm wavelength and its four individual components. This example defines a “favorable” case where all four individual AODs are assessable and the spread between them is quite small (about 0.02 or less). This spread is comparable with the upper limit of expected calibration uncertainty (0.02) and can be attributed to several factors, such as different data processing schemes and design of instruments. As expected, the combined AOD is located between the two individual AODs with largest and smallest values and its diurnal changes follow those of the individual AODs (Fig. 2a).

Good visual agreement between individual time series (Fig. 2a) is supported by the basic statistics provided by the linear regressions applied to six pairs of four individual AODs (Table 2). These AOD pairs are defined here as follows: pair #1 (MFRSR C1 vs. CSPHOT), pair #2 (MFRSR E13 vs. CSPHOT), pair #3 (NIMFR vs. CSPHOT), pair #4 (MFRSR C1 vs. NIMFR), pair #5 (MFRSR E13 vs. NIMFR), and pair #6 (MFRSR E13 vs. MFRSR C1). For example, the square of correlation coefficient (R^2^) exceeds 0.92 for all pairs (Table 2). The slope is moderate (~0.8) and large (~1.0) for the first three and last three pairs, respectively (Table 2). It should be emphasized that sample size for the first three pairs (Np ≤ 44) is about ten times smaller in comparison with that for the last three pairs (Np ≤ 438). Therefore, the difference between slopes obtained for the first and last three pairs can be attributed, at least in part, to the substantial difference between sample sizes. Note, the generated product with both the individual and combined AODs includes these statistics (“Data Records” section).

**Table 1:** Specification of four collocated ground-based instruments at the ARM CF. Number with * indicates an umbral angle of a sky strip blocked by shadowband^44^.

Name	Wavelengths (μm)	Field-of-view (°)	Temporal resolution	Design
Two MFRSRs (C1 and E13)	0.415,0.5,0.615,0.675,0.87,0.94	~3.3*	20 sec	shaded and unshaded data
NIMFR	0.415,0.5,0.615,0.675,0.87,0.94	5.7	20 sec	sun-pointing
CSPHOT	0.34, 0.38, 0.44, 0.5, 0.675, 0.87, 1.02, 1.64	1.2	10–15 min	sun-and sky-tracking

We also assess the temporal variability of the combined AOD for the day considered here (May 11, 2008). For our assessment, we calculate both the range and standard deviation (StDv) of the combined AOD at a given time if at least two overlapped AODs are available. The StDv is specified as an upper limit of expected calibration uncertainty (0.02) if a single AOD is available. It should be noted that the range is twice as large as the StDv when only two individual AODs are available. For the majority of time instances (~80%), the calculated StDv does not exceed 0.01 (Fig. 2b). Here and below time series of the aerosol properties (e.g., individual and combined AODs, StDv) obtained with high (1-min) temporal resolution are used to calculate their daily-averaged counterparts. At least a single 1-min input is required for such calculations. The daily-averaged StDv is small (0.01) at the selected two wavelengths (0.5 and 0.87 µm). This suggests that the time series of 1-min StDv at these two wavelengths exhibit weak temporal variability for this day. The corresponding daily-averaged value (0.58) of Angstrom Exponent calculated for the combined AOD at these two wavelengths is comparable with those obtained for three individual AODs (MFRSR C1, MFRSR E13 and CSPHOT) and underestimates slightly (about 15%) that found for the NIMFR AOD.

The variability assessed above can be viewed as a “practical” uncertainty of the combined AOD. Its “theoretical” counterpart would involve conventional error propagation analysis and assumed uncertainties of individual AODs, which are commonly unknown for a given case. For example, it could be assumed that an upper limit of expected calibration uncertainty (0.02) defines the uncertainty of all individual AODs and four individual AODs with the same uncertainty (0.02) are available from four instruments. In this case, these uncertainties would propagate to a noticeable (0.04) “theoretical” uncertainty of the combined AOD with four individual components. However, a visual inspection of the individual and combined AODs (Fig. 2a) suggests that the uncertainty of the combined AOD should be smaller (roughly by factor of two) and thus, the assumption made about the individual uncertainties (0.02) is not applicable for the case considered here (Fig. 2a). To escape the outlined discrepancies, we use a “practical” uncertainty of the combined AOD and the complementary main statistics describing the level of agreement between its individual components with focus on user-specified needs.

Different applications of the combined AOD may have different user-specified requirements in terms of acceptable uncertainty and thus the corresponding sample size. The statistics of the StDv (Fig. 2b) offer an opportunity to accomplish these goals. For example, the fraction of acceptable time instances (or sample size) of the combined AOD increases from 80% to 95% by increasing the StDv threshold from 0.01 to 0.015 (Fig. 2b). Results from Table 2 and Fig. 2b suggest that single parameter (StDv) can be used for selection of “favorable” cases where the level of agreement between individual AODs can satisfy user-specified needs. In particular, these results suggest that linear regressions for pairs with overlapped individual AODs would have a large (>0.9) correlation coefficient, a “close-to-one” slope, and a small (<0.02) bias when the calculated StDv of the combined AOD is small (0.015 or less). We confirm this suggestion below (“Technical Validation” section) using daily-averaged AODs.

**Table 2:** Parameters of linear regressions obtained for six AOD pairs for a given day (May 11, 2008): number of points (N), square of correlation coefficient (R^2^), slope and mean difference (bias).

AOD pair	N	R^2^	Slope	Bias
1	44	0.952	0.845	0.007
2	38	0.989	0.796	−0.011
3	34	0.986	0.810	−0.013
4	382	0.926	1.01	0.02
5	355	0.984	0.986	0.002
6	438	0.943	1.044	0.019

It is expected that the combined AOD would have an extended temporal coverage in comparison with its individual components. To illustrate, we calculate the total number of time instances of the combined AOD at 500 nm wavelength and its four individual components (Fig. 2c). The total number for the combined AOD surpasses slightly those calculated for two instruments (MFRSR C1 and MFRSR E13) and substantially (up to factor of ten) the total number determined for the CSPHOT (Fig. 2c). The obtained slight (combined vs. MFRSR C1 and MFRSR E13) and substantial (combined vs. CSPHOT) difference is attributed mostly to the fine (MFRSR AODs) and coarse (CSPHOT AOD) temporal resolution of individual AODs.

## Data Records

The combined AOD data with four components considered here are available for a 21-yr period (from 1997-01-16 to 2018-12-05; Table 3). These data have been uploaded to figshare during the submission process^38^. The uploaded data have name (sgpqcaodC1.c1.tgz), title (Quality Control Aerosol Optical Depth Value-Added Product) and the following description: The Quality Control Aerosol Optical Depth (QCAOD) Value-Added Product (VAP) generates sgpqcaodC1.c1 dataset with combined aerosol optical depth (AOD) at two wavelengths (500 and 870 nanometers) with high-quality, enhanced continuity and fine (1-minute) temporal resolution. The tar file sgpqcaodC1.c1.tgz contains the 7965 netCDF files for the sgpqcaodC1.c1 dataset from 1997 to 2018. The uploaded data are stored as time-series; one file per day. File names are selected in accordance with the established ARM file-naming convention. For example, file name “sgpqcaodC1.c1.YYYYMMDD.hhmmss.nc” includes information on year (YYYY), month (MM), day (DD), hour (hh), minute (mm) and seconds (ss). The times in the file are the beginning of the minute. The data format in these files follow the ARM Data File Standard, version 1.2^39^. A full list of the variables in these files is provided by a corresponding technical report^40^.

**Table 3:** General information regarding the combined AOD and its four individual components: data products (first column), name of the corresponding files (second column) and their temporal coverage (third column).

AOD Product	File (datastream) name	Period
Combined	sgpqcaod.c1^45^	1994-04-07 to 2018-12-05
MFRSRs (C1 and E13)	sgpmfrsraod1michC1.c1^46^	1997-01-16 to 2018-12-05
	sgpmfrsraod1michE13.c1^46^	1997-01-16 to 2018-12-05
NIMFR	sgpnimfraod1michC1.c1^47^	2000-05-03 to 2017-12-14
CSPHOT	sgpcsphotaodfiltqav3C1.a1^48^	1994-04-07 to 2019-10-06

Note, time periods specified by the ARM convention, in general, are inclusive on the start date and exclusive on the end date. Included references (second column) provide important details and ARM archive links.

For an earlier period (from 1994-04-07 to 1997-01-15, inclusive), the combined AOD data contain only the CSPHOT AODs (Table 3), and they are not considered in our paper. To access the generated AOD products uploaded to the ARM Archive (Table 3), an ARM account with free registration is required. These AOD products are freely available to any users that create an ARM account.

## Technical Validation

**Fig. 3:**
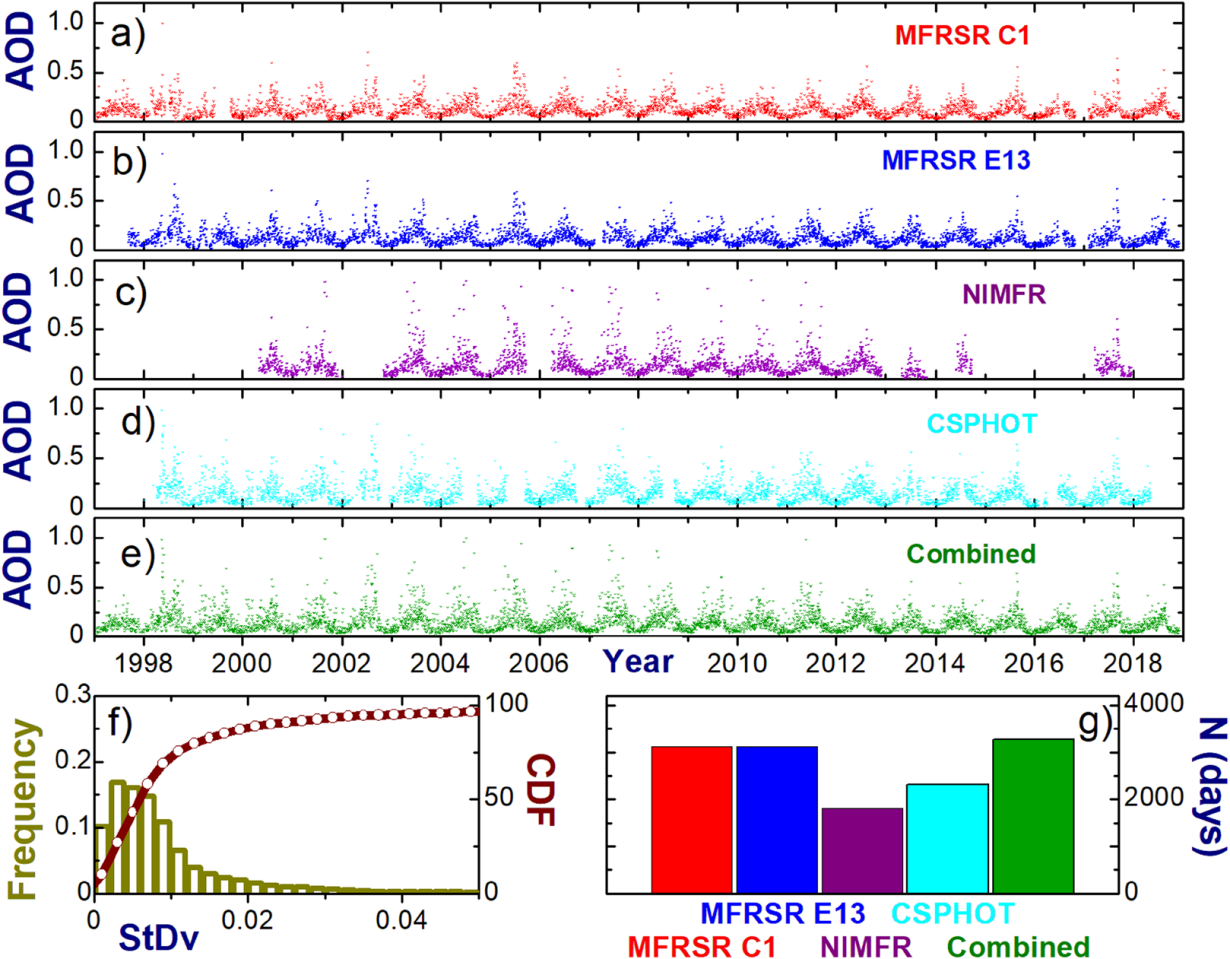
Demonstration of how to combine the strengths of the individual daily-averaged AODs in a complimentary way for the 21-yr period considered here. (**a**–**e**) temporal changes of the individual and combined AODs, (**f**) normalized frequency and cumulative distribution function (CDF) of standard deviation (StDv) of the combined AOD, (**g**) the number of days of the individual and combined AODs where StDv is small (<0.01).

To further demonstrate technical quality of the dataset, we consider the daily-averaged values of the individual AODs (Fig. 3a–d) and the related outputs (Fig. 3e–g). Time series of the individual AODs exhibit seasonal patterns with large and small AOD values during summer and winter, respectively (Fig. 3a–d). The strong seasonal variability of AOD at the SGP CF has been documented previously^21,22^. Note, these time series (Fig. 3a–d) passed successfully automated QC tests. However, sometimes (e.g., summer of 2003), the NIMFR AODs obtained at 500 nm (Fig. 3c) exceed substantially those provided by the other three instruments, suggesting that these NIMFR AODs are “incorrect” and likely cloud-contaminated or suffer from sun-tracking problems. Thus, the automated QC tests, which are very useful for preliminary analysis of data quality, occasionally are not able to detect “incorrect” data.

Similar to the individual AODs, the combined AOD has a distinct seasonal pattern with large (during summer) and small (during winter) values (Fig. 3e). Also, the combined AOD has occasional “incorrect” values due to unscreened “incorrect” NIMFR AODs. Events with “incorrect” AODs are rare (less than 3% of time) and represent large (>0.05) values of the StDv of the combined AOD (Fig. 3f). Thus, an appropriate selection of the StDv threshold can easily remove these “incorrect” AODs. For example, by choosing a small (0.01–0.02) threshold one can preserve the outlined distinct seasonal pattern of the combined AOD but eliminate days with “incorrect” AODs. It should be emphasized that the StDv does not exceed the prescribed uncertainties (0.01–0.02) of the individual AODs for the majority of cases (~90% of time). Other potential options for removing the “incorrect” AODs could include selection of maximum thresholds of the combined AOD and conventional two-sigma interval (e.g., 1-min data). However, these options are not considered here.

Time series of the individual AODs (Fig. 3a–d) have gaps, which are likely associated with the instrument-dependent data quality issues and the required upgrades and replacements of these instruments. Duration of these gaps is instrument-dependent and can be substantial (from several weeks to several months). The NIMFR AOD record with many gaps has the longest durations of gaps (up to several years) compared with the AOD time series from the other three instruments. In contrast, the MFRSR AOD records show the best continuity. To illustrate an extended coverage of combined AODs with good quality, we include an example (Fig. 3g) which defines a small (0.01) value of the StDv threshold. This example demonstrates two main points. First, coverage of the combined AOD is comparable with those obtained for two MFRSR AODs mostly due to their greater continuity compared to AODs provided by the other two (NIMFR and CSPHOT) instruments. Second, temporal coverage of the combined AOD is substantially greater than that obtained from the NIMFR and CSPHOT AODs. This coverage difference is about 80% and 40% for the NIMFR AOD and CSPHOT AOD, respectively. Several factors, such as large gaps in the NIMFR AOD and CSPHOT AOD data sets, and their partial overlaps (Fig. 3c,d), have an impact on this difference.

Results obtained for a given day (Fig. 2, Table 2) suggest that the StDv of the combined AOD can be used for selection of favorable cases where all individual AODs are in a good agreement. To confirm this suggestion, we include the basic statistics of the linear regressions between individual daily-averaged AODs for two values (0.01 and 0.02) of the StDv threshold (Tables 4, 5). These statistics illustrate that level of agreement is a quite high and depends weakly on the chosen two StDv values at 500 nm wavelength. Similar reasonable agreement and weak dependence on the selected threshold is obtained at 870 nm. Therefore, application of the spectrally-independent threshold (e.g., ≤0.02) can be used to identify days where all individual AODs are quite consistent with each other.

**Table 4:** Parameters of linear regressions obtained for six pairs of daily-averaged AODs at 500 nm wavelength for 21-year period (1997–2018) and small (0.01) value of the StDv threshold: number of days (N), square of correlation coefficient (R^2^), slope and mean difference (bias).

AOD pair	N	R^2^	Slope	Bias
1	2184	0.973	0.967	−0.003
2	2201	0.974	0.949	−0.002
3	1363	0.981	0.973	0.003
4	1727	0.986	0.997	−0.007
5	1721	0.988	0.992	−0.005
6	3006	0.989	0.978	0.001

**Table 5:** The same as Table 4 except for different value (0.02) of the StDv threshold.

AOD pair	N	R^2^	Slope	Bias
1	2865	0.951	0.942	−0.001
2	2890	0.950	0.920	0.001
3	1860	0.953	0.950	0.005
4	2302	0.979	1.000	−0.008
5	2299	0.981	0.991	−0.006
6	3812	0.984	0.972	0.001

## Usage Notes

For user convenience, the individual AODs are supplemented by information on the data quality and the pairwise consistency of the available individual AODs for each day. Note, there are six pairs when four individual AODs are available for a given instance. Because the pairwise comparisons are only one way of evaluating these data, we encourage potential users to consider additional complimentary information on the variability of the combined AOD; here the term “variability” refers to the spread between the individual AODs for a given instance where they overlap. By choosing an appropriate threshold for this variability, a user can control the consistency for each pair. For example, we have demonstrated that the pairwise consistency is good (e.g., R^2^ is close to 1) when the variability threshold is specified to match the expected uncertainties (0.01–0.02) of the measured AODs. In other words, application of this single parameter allows a user to skip examination of many parameters, which represent several pairwise comparisons (up to 18 parameters for six pairs). Certainly, the required level of pairwise consistency depends on user-specified needs and an appropriate selection of the StDv threshold can address these needs easily. Such selection could include a wavelength-dependent threshold and/or its normalized version if needed. For example, the mean value of the combined AOD can be used for obtaining the normalized StDv threshold (StDv/Mean) for a given day of interest.

The integrated two-decadal dataset successfully leverages the accuracy of individual AODs, their long time period, and high temporal resolution. Thus, this dataset with a straightforward data format and public access allows a user to analyse variability of aerosol loading over multiple time scales and to demonstrate the importance of this variability in forcing the climate change. In particular, this dataset can be aggregated to coarser time scales (e.g., daily, monthly or annual averages) to be consistent with those of model predictions and other data products relevant to user interest. The merging of this dataset with other products provides the opportunity to give a comprehensive picture of how aerosol loading can be tied together with specific climate-related factors. For example, the prospective merging of this dataset with wildfire products^41^ would make it possible to correlate the AOD changes with wildfire history, and thus to improve understanding of the relationship between wildfires and aerosol burdens^42^. Furthermore, the merging of this dataset with surface radiation products would be beneficial for reducing uncertainties of radiative forcing at different time scales due to aerosol perturbations^32,43^ and for radiative closure experiments with the main goal to determine the precision with which a radiative transfer model can predict both direct and diffuse irradiances at fine temporal scales^29,30^. It should be mentioned that extensive data on aerosol, cloud and meteorological properties, and surface radiation are available at the ARM SGP CF^20^. Therefore, these data are well suited for process-oriented studies^22,27^.

## Data Availability

The code used to generate the combined AOD product (sgpqcaod.c1) is available on Github (https://github.com/ARM-Development/qc_aod). All computations have been performed using the Python environment (v3.6.8) and the ARM Data Integration (ADI) library (https://github.com/ARM-DOE/ADI). More information about ADI is available at the ADI Documentation website (https://engineering.arm.gov/ADI_doc/index.html).
